# Lessons Learned From Building a Global Health Partnership on Obstetric Care in Madagascar

**DOI:** 10.9745/GHSP-D-22-00521

**Published:** 2023-10-30

**Authors:** Caroline Benski, Monica Zambruni, Giovanna Stancanelli, Tsiriniaina Landinarisoa, Abéline Hantavololona, Vonimboahangy Rachel Andrianarisoa, Paulin Ramasy Manjary, Cecilia Capello, Begona Martinez de Tejada, Michael R. Reich, Anya Levy Guyer

**Affiliations:** a Département de la femme, l'enfant et l'adolescent, Hôpitaux Universitaires de Genève, Geneva, Switzerland.; b Terre Innovative, Catania, Italy.; c Projet Santé de la Reproduction, Ministère de la Santé Publique, Antananarivo, Madagascar.; d Service Maternité Sans Risque, Direction de la Santé Familiale, Ministère de la Santé Publique, Antananarivo, Madagascar.; e Inspection de la Santé du District d'Ambanja, Ambanja, Madagascar.; f Enfants du Monde, Geneva, Switzerland.; g Department of Global Health and Population, Harvard T.H. Chan School of Public Health, Boston, MA, USA.; h Independent consultant, Boston, MA, USA.

## Abstract

The authors share experiences of a global health partnership that worked to promote equity through a commitment to shared values and goals, engagement and communication, and mutual trust and respect.

## Key Messages

- We share experiences of a global health partnership between 2 hospitals, the Ministry of Health, and a nongovernmental organization that focused on improving obstetric care at a district hospital in northwestern Madagascar.
- Despite having differing access to material, technical, and financial resources, the partners cultivated an environment for equity through a commitment to shared values and goals, active engagement in communication and implementing activities, and fostering of mutual respect and trust among individuals and institutions.
- The success of the partnership also depended on flexibility and a willingness to use technology in new ways, especially when COVID-19-related restrictions necessitated the adaptation of a simulation-based training activity on respectful emergency obstetric and neonatal health care.

## INTRODUCTION

A global health partnership is a collaborative effort among 2 or more institutions that agree to work together toward a common public health goal. The types of institutions involved in global health partnerships vary widely. They may include government agencies, academic and research institutions, entire health systems, individual hospitals and clinics, businesses, nongovernmental organizations (NGOs), and others.^1^^,^^2^ Most global health partnerships link institutions in the Global North and the Global South, and some have achieved notable success in reaching tangible and mutually agreed goals.^3^^,^^4^

However, significant concerns persist about asymmetrical power dynamics in global health partnerships.^5^ These dynamics occur particularly when a partnership involves institutions with unequal access to resources (including status).^6^ When institutions from the Global North are involved in global health partnerships, they typically wield significantly more power than organizations from the Global South. This is partly due to better access to funding, training, equipment, specialist expertise, and other resources. Equally important, power dynamics in global health partnerships are often predicated on and replicate sociocultural assumptions and practices with deep roots in colonialism, neocolonialism, and racism, along with other forms of inequity and injustice.^7^^–^^9^ Recently, the phrase “decolonizing global health” has been increasingly used to describe efforts to transcend traditional power dynamics and build more equitable relationships among stakeholders in global health.^10^

In this commentary, we reflect on our experiences creating a 4-institution global health partnership with a programmatic focus on improving obstetric care at a district hospital in northwestern Madagascar. In addition to implementing effective programming on obstetric care, we consciously worked to foster equity among the partners despite our varied access to technical and financial resources. In retrospect, we identified 3 elements of our approach to partnership that supported our desire for equity: (1) commitment to shared values and goals, (2) active engagement in communicating and implementing activities, and (3) fostering of mutual trust and respect. No single path exists for effectively decolonizing global health partnerships, and to some degree, each partnership will be different. This commentary presents our lessons learned to support other global health partnerships seeking to decolonize their activities through respectful and equitable collaboration.

## GLOBAL HEALTH PARTNERSHIP TO IMPROVE EMERGENCY OBSTETRIC CARE

Centre Hospitalier de Référence du District d'Ambanja (CHRD) in Madagascar is the district hospital for Ambanja district in northwestern Madagascar. It has 100 beds and approximately 1,000 deliveries per year. Access to quality obstetrical care is a major challenge throughout the country, particularly in emergency situations. Most pregnant women living in Ambanja deliver at home, so those who come to CHRD tend to be high risk or in need of emergency obstetric and intensive post-delivery care. Providing quality care for pregnant women and newborns in Ambanja district is a difficult challenge because the hospital is chronically under-resourced and understaffed. In Madagascar, district hospitals fall into a resource gap—most of the limited funding and training opportunities that exist go to either community-based primary care centers or hospitals in the capital. However, despite these challenges, the CHRD staff were committed to providing quality care to the best of their abilities, with limited supplies and equipment.

For more than 8 years, CHRD maternity department staff communicated with a colleague in Switzerland and developed a relationship of trust. This obstetrician/gynecologist at Hôpitaux Universitaires de Genève (HUG) had previously spent a year working on cervical cancer and mobile health projects in Ambanja.^11^ HUG has the largest maternity department in Switzerland, managing more than 4,000 deliveries per year. The HUG system is involved in various international collaborations, with partners in more than 20 countries and strong connections with the World Health Organization and international NGOs.^12^

In 2019, physicians at CHRD requested training for maternity department staff on emergency obstetric and neonatal care, and the HUG physician and her colleagues in Switzerland agreed to respond. This was the first step in building our partnership for improving emergency obstetric care in Ambanja. Extensive discussions ensued between the hospital teams. As they discussed ideas for collaboration on this topic, it became clear that their efforts would benefit significantly from additional support from other institutions. Two key institutions were approached that then became core members of the partnership: the Ministry of Health (MOH) in Madagascar and the Swiss NGO Enfants du Monde, an NGO with expertise in training providers in low-income countries on respectful maternal care. Other institutions have also made significant contributions to the partnership's work, including an Italian social enterprise, Terre Innovative, and several technical and donor agencies.

As staff from the 2 hospitals discussed ways to collaborate, it became clear that their efforts would benefit significantly from additional support from other institutions.

Through discussions, the 4 institutions agreed on a plan to conduct a simulation-based integrated training on respectful emergency obstetric and neonatal care. Simulation is a mode of instruction that uses mannequins, actors, and group role-plays to replicate clinical situations for training purposes.^13^^,^^14^ Participating in simulations allows trainees to practice both clinical skills and team collaboration in a controlled environment where they receive constructive feedback and support. This type of simulation-based training has had significant success in emergency obstetric and neonatal care^15^^–^^17^ but had not previously been used in northern Madagascar.

Implementing the training required the involvement of several staff from each partner and additional funding to support the activity. On behalf of the partnership, HUG applied for a grant of approximately 100,000 Swiss francs (US$100,000) from the (now closed) Swiss branch of the ESTHER Alliance for Global Health Partnerships. The partnership structure was then formalized by creating and signing a multilateral agreement that detailed each partner's roles and responsibilities. The simulation-based training on respectful emergency care for women and newborns was implemented in 2021. The training took place in a designated room at CHRD that was equipped with Internet, computers, a screen, smartphones, and other technology that enabled the Swiss partners to participate fully but remotely.

This short summary glosses over many challenges. Some were typical challenges faced in any situation when organizations with different missions and goals try to work together. Others were more specific to the sudden appearance of COVID-19, which drastically affected our original plans for implementing the training.^18^ In response to the pandemic, each partner institution had to rapidly and radically adapt its operations. These shifts had numerous implications for the partnership's activities. For example, to adapt to the lack of in-person visits, the partners tested and embraced technological solutions. These allowed us to continue communicating and collaborating and enabled the Swiss team and partners based in Antananarivo to participate remotely in the training.

Three other major changes were required to adapt to the COVID-19 pandemic. First, because the Swiss partners could not travel to Madagascar, we added a training-of-trainers component for MOH staff. Another adaptation was conducting the training twice for 2 smaller cohorts of CHRD staff (instead of once for the full CHRD team) to limit potential COVID-19 exposures. The third major change related to the impossibility of getting certain materials used in simulations delivered to Ambanja. Workarounds included replacing high-tech mannequins with low-tech models, adding additional role-playing by the trainers, and designing training materials that could be produced in Ambanja. Each of these changes ultimately had notable benefits for the partnership. The training of trainers built national capacity and support for the use of simulation. The smaller training cohorts gave each trainee more opportunities to participate. Finally, adapting the required materials to the local context made the simulations more realistic and easier to replicate in the future.

While evaluation of the impact of the training is ongoing, our early analysis of forthcoming results looks excellent (unpublished data). Among our emerging findings were: (1) the hybrid format and the course content were well-received by the CHRD participants; (2) the trainees' scores on tests of knowledge about the management of obstetric emergencies improved; and (3) most participants reported improved communication with their colleagues and patients.

Following the training, the partners reflected on this first major partnership initiative to examine whether and how it worked. The reflection involved an inductive process to identify key elements that contributed to the partnership, self-assessments of the partnership using ESTHER's “EFFECt Tool,” and a comparison of our conclusions with the results of other partnership frameworks.^19^^–^^21^

## ELEMENTS OF A STRONG PARTNERSHIP

We concluded that our partnership has succeeded because of 3 elements: (1) a commitment to shared values and goals, (2) active engagement, and (3) mutual trust and respect. Each of these elements exists both at the institutional and individual levels (Figure).

**FIGURE.**
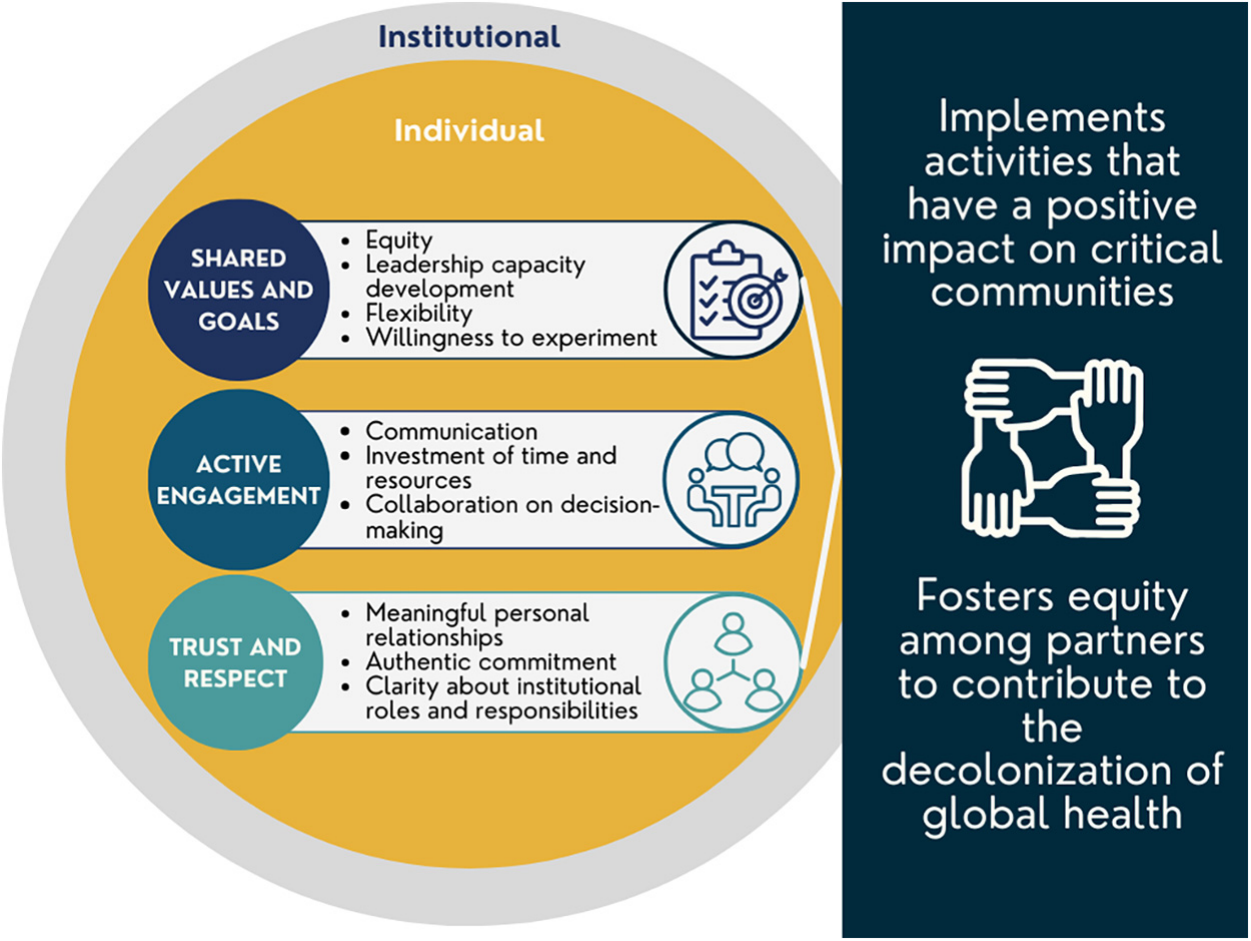
Elements of a Strong Global Health Partnership

We did not consciously focus on these 3 elements at the start of the partnership, nor did we explicitly articulate a desire to contribute to the decolonization of global health. However, having identified the elements, we now actively discuss how to sustain these elements during planning conversations. We continue to explore good practices in communications, as well as for planning, implementing, and evaluating activities. In this section, we present our operating definitions of these features of the partnership.

### Commitment to Shared Values and Goals

Each of the 4 partner institutions is a different type of institution working in a different context. Therefore, to collaborate effectively, each institution had to commit to shared goals and values related to the partnership's programmatic objectives and collaborative process. As a partnership, we committed to the goal of building the capacity of CHRD to provide quality maternal health care. We also created shared values to guide the strategies we used in working toward our goal. In hindsight, we identified the shared values of the partnership.

- Each institution (and individual) conscientiously fulfills the commitments it makes to the partnership.
- The ideas, opinions, and concerns of all partnership members are taken seriously.
- Each institution and its individual representatives participate in good faith in collaborative and consensus-oriented decision-making.

The partners created shared values to guide the strategies used to achieve the goal of improving the quality of obstetric care at CHRD.

Many of our partnership's operating approaches emerged organically based on the personalities of and relationships among the individuals involved. Others were articulated in the institutional agreement and other documents.

A key component of our communication and decision-making processes was that all partners listened carefully, especially to opinions expressed by the CHRD team. The CHRD team had expertise on the conditions in Ambanja, so their understanding of what would be useful and applicable in that context was considered by all partners to be of paramount importance. Thus, in the discussions for planning the training, they were explicitly asked to provide their input, which was given extra weight during decision-making.

Another shared value was transparency around bureaucratic and financial management.^22^ Each partner institution articulated its financial and administrative requirements so that other partners understood their separate needs and limitations. Malagasy partners were provided stipends in line with the MOH standard for their work on the training, while the Swiss partners received no additional salary or stipend. HUG endeavored to communicate clearly about the financial and administrative processes required by the funding agency, ESTHER, including sharing the forms and reporting requirements. Sharing information about bureaucratic processes helped build mutual understanding and capacity across the institutions.

Finally, the partners shared a willingness to be flexible and experiment with new technologies and methodologies. Some approaches that now feel central to the partnership's work (such as the use of a hybrid in-person and remote training methodology) were not in our original plans. Instead, these strategies evolved as we worked closely together to implement the simulation-based training within the limitations and obstacles created by the COVID-19 pandemic. The partnership adapted implementation strategies in response to changes in the environment by updating and adding goals, testing various technologies and methods for communication, and using new tools. All partners were as committed to adaptation, change, and evolution as they were to the original goal of supporting CHRD to improve the quality of obstetric care.

### Active Engagement in Partnership Activities

This element encompasses the significant investments of staff time and other resources made by each institution. Although each institution officially assigned 1 or more focal point persons to the partnership, in reality, numerous staff members were involved in the partnership's activities.

One important component of active engagement was the willingness of all partners to engage in frequent and multichannel communications. Collaboration is a dynamic endeavor, particularly in a rapidly changing environment. Regular communication was required during the design, adaptation, implementation, and evaluation of the training and ad hoc activities. We used various modes of communication for these dialogues, including telephone calls, videoconferences, and chat groups. Written chat threads (on WhatsApp and the Messenger app) were especially important because they allowed all institutional representatives to follow and remain current on programmatic and administrative dialogues in real-time or asynchronously.

One important component of active engagement was the willingness of all partners to engage in frequent and multichannel communications.

Active engagement was especially evident at the individual level. Many individuals did significant partnership-related work outside of their regular working hours. This required high levels of motivation (which can be difficult to sustain, especially in the face of delays or when in-person participation is not possible). Maintaining individual motivation at times was challenging and sometimes required reallocating tasks to other individuals.

### Mutual Trust and Respect

The third element that contributed to success was the trust and respect shown by each institution and individual. The partnership initially emerged out of interpersonal relationships grounded in trust and respect.^21^^,^^23^ For us, “trust” meant operating with the assumption that each partner spoke and acted with sincerity and honesty; similarly, “respect” meant valuing the input of each partner while acting sincerely and honestly.

As the partnership grew, trust and respect continued to be earned and offered by each institution and individual. We did this by demonstrably committing to and actively engaging in the work of the partnership—that is, through the other 2 key elements. If 1 partner demonstrated engagement in the partnership's work, then other partners accorded them respect by honoring their contributions, soliciting their input, and supporting capacity development.

Trusting the local knowledge of each team was also important. For us, this meant relying on CHRD to determine which training topics and modalities would work best for their staff, on the MOH to provide guidance on national standards, and on the Swiss partners for training expertise and adherence to the funder's requirements. Trusting other partners was not always easy. Each partner, at some point, had to relinquish expectations, give up control of certain tasks, and accommodate other institutions' procedures.

In the absence of mutual trust and respect, a partnership is unlikely to work. Lack of trust undermines the capacity to collaborate. When partners feel distrusted, they will resist sharing concerns; when they feel disrespected, they will not share ideas. When trust and respect are withdrawn or lost, it can have huge consequences; if these ruptures are not addressed, it can lead to the end of a partnership. Thus far, our partnership has been both lucky and careful to sustain mutual trust and respect. When we consider new initiatives (and as new staff members are added to the partnership), we work to continually reinforce trust and respect through authentic and active communication and engagement.

If loss of trust and respect is not addressed, it can lead to the end of a partnership.

### Two Levels: Institutional and Individual

Our experience shows that the elements of successful partnership must exist at both the institutional and individual levels. Investments of time, energy, and resources are essential from both individuals and the institutions they represent. Each institution must fulfill commitments and follow through on promised investments in the partnership, including participation in collaborative decision-making. To do this, individuals within each institution must undertake the work required and receive appropriate support from their institution.

The attitudes and behaviors of individuals can make or break a partnership; similarly, the values and policies of institutions must be sufficiently aligned to make collaboration equitable and effective. Trust and mutual respect are especially critical at the individual level. The collegial relationships among the founding individuals in our partnership enabled direct, realistic, and open communication, setting a precedent for the partnership's approach to decision-making. In particular, as the partnership grew, the Swiss partners' responsiveness to requests from Madagascar demonstrated respect for the Malagasy colleagues. In turn, this respect solidified the Malagasy partners' trust in the Swiss partners' commitment to equity.

### The Challenge: Sustaining Key Elements Through Institutional Changes

Change is inevitable, and our partnership is grappling with how to sustain the core elements of success as we expand, adapt to institutional changes, and respond to external circumstances. We were lucky that only a few key individuals in the partnership left their posts during the planning and implementation of the emergency obstetric care training. In each case, those who left carefully selected and oriented a replacement to take over as the partnership's focal point person.

We learned from that experience that we need to develop more systematic ways to sustain the partnership through gradual and sudden changes. We have begun systematizing how we orient new participants to the partnership's goals, values, and approaches to collaboration; shift roles and responsibilities as individuals move to different positions; share the partnership's past experiences and values; remain open to input and new opportunities; and build mutual trust and respect among longstanding and new members. We are dealing with these issues as we explore expanding the partnership's work into new areas, including building capacity at CHRD to routinely test for Rh-factor and testing and treating pregnant women for HIV and other sexually transmitted infections.

## LESSONS LEARNED

Our partnership represents a “north-south” engagement that has successfully implemented an initial project—simulation-based training—that contributes to a shared long-term goal to improve the quality of obstetric care in Ambanja. Despite the complexity of building a new partnership and in the face of complications created by the COVID-19 pandemic, we were able to achieve this goal through our commitment to shared values, active engagement, and mutual trust and respect. The organic evolution of this partnership may not be easily replicated by others, but we offer 4 overarching lessons we have learned from our experience.

- Building long-term collaborative relationships among institutions with similar missions but different approaches and resources can have significant positive impacts, as shown by our efforts to improve the quality of maternity care at CHRD.
- A strong partnership depends on 3 critical elements: clearly stated shared values and goals, active engagement, and trust and respect among partners.
- Partnerships exist on multiple levels, but they must especially involve both institutions and individuals in the 3 critical elements.
- The personal relationships and institutional structures that make a partnership work are complex and require transparency, communication, appreciation, and responsiveness.

We hope that these lessons are useful for others working to build equitable global health partnerships, decolonize global health, and contribute to improving health outcomes globally.

## Notes

**Peer Reviewed**

**First published online:** September 28, 2023.
